# The Light-Treatment of Disease

**Published:** 1903-07-04

**Authors:** 


### THE LIGHT-TREATMENT OF DISEASE.

It has been known from very early times that light exercised an important action on the well-being of the human body, and many physicians seem to have had a glimmering knowledge that different colours affected the body in different ways. The classical example is that of John of Gaddesden in 1357, who treated Prince Lionel by wrapping him in scarlet. He says in the "Rosa Anglica," "When the son of the illustrious King of England (Edward II.) had the small-pox I took care that everything about his couch should be red, and his cure was perfectly effected, for he was restored to health without a trace of disease." The superstition was world-wide, and is only a part of the doctrine of signatures which still flourishes in many remote parts of England where the white witch thrives at the expense of the doctor. In China red silk is still braided in the hair of children, and in New Zealand red was used liberally at death; the idea in these cases being that red is obnoxious to evil spirits. General Pleasonton in America, Alcina and Auber in Spain, gave an impetus to the light-treatment of disease, whilst Charcot's experiments at the Salpetrière did something to put it on a more scientific footing. The discovery of the Röntgen-rays led to further improvement, which was skilfully taken advantage of by Dr. Finsen of Copenhagen. Dr. Finsen's name is associated with two different forms of phototherapy, the treatment of the exanthemata, especially small-pox, by the use of rays derived from the violet end of the spectrum, and the cure of certain diseases of the skin, such as rodent ulcer and lupus. The rays obtained from the chemical end of the spectrum—that is to say, from the blue, violet, and ultra-violet portions—are the most active, for these rays are capable of causing an inflammation or sunburn when the skin is exposed to their action. The blue and violet rays are excluded, and the ultra-violet portion of the spectrum is now relied upon to effect a cure in appropriate cases. The ultra-violet rays are found naturally in sunlight, and they may be produced artificially by an electric arc-light or by an electric spark from a condenser. They have interesting physical and physiological properties. They are powerfully actinic, they excite fluorescence, they can discharge a body which is negatively electrified, and they can produce nuclei for cloud condensation in moist air. Physiologically, they have a bactericidal power, they are able to produce an inflammation of the skin, and they can exert their action to some depth in animal tissues because they have the power of penetration. This was prettily shown by Dr. Godneff some years ago. He placed small sealed glass-tubes containing silver chloride under the skin of animals, some of whom he afterwards kept in the dark and others in sunlight. He took the tubes out at the end of an hour to find that the silver salt was reduced in the animals exposed to sunlight, whilst it remained unaltered in those which had been kept in the dark. Dr. Finsen further proved that the ultra-violet rays can penetrate bloodless tissues much more easily than those which are filled with blood. They pass with difficulty through glass or mica, but easily through the air, rock-salt, quartz, and ice. The rays are obtained either from an arc lamp—which is inconvenient even when iron instead of carbon electrodes are employed, as in Dr. Bang's lamp, for iron is very rich in violet and ultra-violet rays—or by the Gorl lamp, which makes use of the condenser spark on account of the ease and simplicity with which it can be obtained and regulated. The open end of the lamp is provided with a piece of rock-salt or ice, which is pressed against the tissue to be influenced in order to make it anæmic. The light-treatment is especially satisfactory in cases of lupus vulgaris and to a lesser extent in lupus erythematosus. It has been employed with very good results in rodent ulcer, acne, and alopecia areata; but it has proved useless in the treatment of favus, ringworm, and sycosis, and it does not cure cancer, though it puts an ulcerating surface into a more healthy condition. The great objections to the light-treatment at the present time are the expensive apparatus which must be used, the length of time during which the patient remains under treatment, and the skilled assistance which is necessary throughout. In tuberculous lupus, where it is most useful, there is no certainty of permanent cure; for relapses are not uncommon and they are the more likely to occur when there are tuberculous lesions elsewhere in the body which can act as foci of fresh infection to the skin or mucous membranes. The cases must therefore be selected with some care and they must be watched after the cure is completed so that any later manifestations of disease may be treated at once.

